# A pendant awn phenotype linked to the *EMBRYONIC FLOWER 1 LIKE* (*EMF1L*) gene in barley

**DOI:** 10.1007/s00122-025-05105-5

**Published:** 2025-12-19

**Authors:** Srijan Jhingan, Zsa Zsa Friederique Boyny, Twan Rutten, Axel Himmelbach, Nils Stein

**Affiliations:** 1 Leibniz Institute of Plant Genetics and Crop Plant Research (IPK) OT Gatersleben, Corrensstr 3, 06466 Seeland, Germany; 2 Department of Plant Breeding, Justus-Liebig Universität Giessen, Heinrich-Buff-Ring 26-32, 35392 Giessen, Germany; 3 Crop Plant Genetics, Institute of Agricultural and Nutritional Sciences, Martin-Luther-University of Halle-Wittenberg, Halle (Saale), Germany

## Abstract

**Key message:**

*HvEMF1L* mutants display pleiotropic defects in spike morphology, especially awn development in barley. The study integrates mutagenesis, genetic mapping and sequencing technologies for trait dissection

**Abstract:**

Awns are apical extensions of the lemma that are prevalent in many wild and cultivated grass species. In cultivated cereals they are relevant, given their influence on grain yield through contributions to photosynthesis, transpiration, carbohydrate accumulation and drought stress mitigation. While several genetic factors controlling barley awn traits have been identified, their complex network and interactions are not fully understood. This study characterizes a “pendant awn” mutant derived from an ethyl methanesulfonate (EMS)-mutagenized population of the two-rowed winter barley cultivar ‘Igri’ which is marked by shorter, thinner, and less upright awns. Segregation analyses in an F₂ population of 149 individuals indicated a monogenic recessive inheritance of the mutant trait. Molecular genetic mapping identified a 356.8 Mbp region on chromosome 3H associated with the pendant awn mutant trait. Whole-genome re-sequencing of mutant and wild-type individuals led to the discovery of a premature stop codon mutation specific to the pendant awn mutant in the *EMBRYONIC FLOWER 1 LIKE* (*HvEMF1L*) gene, predicted to truncate the protein by 62.3%. Pan-genomic and -transcriptomic analyses revealed a high level of sequence conservation of *HvEMF1L* in global barley germplasm and an inflorescence-specific gene expression profile. The study highlights *HvEMF1L* as a putative regulator of barley awn development and underscores the scope of combining mutagenesis, genetic mapping, and genome sequencing for trait dissection.

**Supplementary Information:**

The online version contains supplementary material available at 10.1007/s00122-025-05105-5.

## Introduction

Awns are stiff apical extensions of the lemma, characteristic of many wild and cultivated grasses including cereals like wheat, rye, oats, sorghum, rice, and barley. They are modified leaves with triangular cross sections, containing two chlorenchymatous and three vascular tissue zones, possess stomata on the abaxial side, and are capable of photosynthetic activity. Although in the wild, awns deter foraging due to their barbed and rough textures, aid in seed dispersal and anchoring in the soil (Elbaum et al. 2007; Guo and Schnurbusch 2016; Huang et al. 2021a, b; Yuo et al. 2012), these aspects are less relevant for today’s cultivated cereals. Nevertheless, awns are important as they can increase assimilation of carbohydrates during grain filling through their photosynthetic ability and mitigate drought stress by increasing water use efficiency (DeWitt et al. 2023; Huang et al. 2021a, b; Patterson et al. 2025).

Awn development in cereals is regulated by a complex and partially conserved network of genes. In rice, multiple key genes have been identified that regulate awn morphology, like *AWN1* (*An-1*) and *AWN2* (*An-2*) that promote awn elongation by stimulating cell division and expansion via cytokinin biosynthesis (Gu et al. 2015; Luo et al. 2013). Genes like the *LONG AND BARBED AWN1* (*LABA1*), *GRAIN LENGTH AND AWN DEVELOPMENT1* (*GAD1*) and *REGULATOR OF AWN ELONGATION* (*RAE*) modulate awn shape and length (Furuta et al. 2015; Hua et al. 2015; Jin et al. 2016). Rice floral meristem regulators including *SHOOTLESS2* (*SHL2*), *SHOOT ORGANIZATION1* (*SHO1*), and *SHO2* affect lemma and awn formation, indicating that proper inflorescence meristem patterning is essential for awn identity (Itoh et al. 2000). In wheat, awn suppression is largely governed by dominant loci such as *HOODED* (*HD*), *TIPPED1* (*B1*), and *TIPPED2* (*B2*) loci (Huang et al. 2020). In barley, *HvKNOX3* is the homolog of *HD* and causes a homeotic transformation of the awn into a second floret in the *hooded lemma1 (kap1)* mutant (Yoshioka et al. 2017) and *LKS2* encoding a *SHORT INTERNODES* (*SHI*)-type transcription factor controls the number of longitudinal cells in the awn (Yuo et al. 2012). *HvMADS1*, which is also functionally conserved in wheat, regulates awn length and lemma width in barley by promoting lemma cell proliferation through cytokinin homeostasis (Zhang et al. 2024a). Moreover, genes involved in the biosynthesis of brassinosteroid have been associated with awn length combined with pleiotropic effects on plant height and leaf and spike morphology (Dockter et al. 2014). Altogether, these studies show that awn development in grasses is under a complex genetic control, integrating cell division, meristem identity and hormonal regulation.

In *Arabidopsis thaliana*, EMBRYONIC FLOWER 1 (EMF1) is a well-characterized developmental regulator and chromatin modulator of floral architecture, repressing premature flowering and maintaining indeterminate shoot growth (Aubert et al. 2001; Shu et al. 2024). Similarly, in rice EMF1-like proteins regulate floral organ identity through epigenetic silencing of several genes (Li et al. 2018; Yan et al. 2015; Zheng et al. 2015). In barley, the *sca* mutant defined by its “short and crooked awns” is documented in the International Database for Barley Genes and Barley Genetic Stocks—https://bgs.nordgen.org/ (Druka et al. 2011; Hansson et al. 2024). Recently, it was shown to carry a mutation in the *EMBRYONIC FLOWER 1 LIKE* (*HvEMF1L*) gene in barley (Nakamura et al. 2025). Independently, we identified a “pendant awn” mutant from an EMS-mutagenized population of the winter-type barley cultivar Igri marked by thin and drooping awns, harboring a distinct premature stop codon mutation in the same gene. Our mutant represents a novel allele and exhibits several effects on spikelet organ identity, providing complementary insights into *HvEMF1L* function. Furthermore, using diversity analyses across global barley germplasm (Jayakodi et al. 2024, 2020; Milner et al. 2019) and pan-transcriptome screening (Guo et al. 2025), we noted high sequence conservation and a general upregulation of *HvEMF1L* in the inflorescence. Taken together, these findings underline its functional constraint and vital role in controlling inflorescence identity and development.

## Results

### Reduced awn length is the most prominent phenotype in the barley EMS mutant M4IGRI_11

**Fig. 1:**
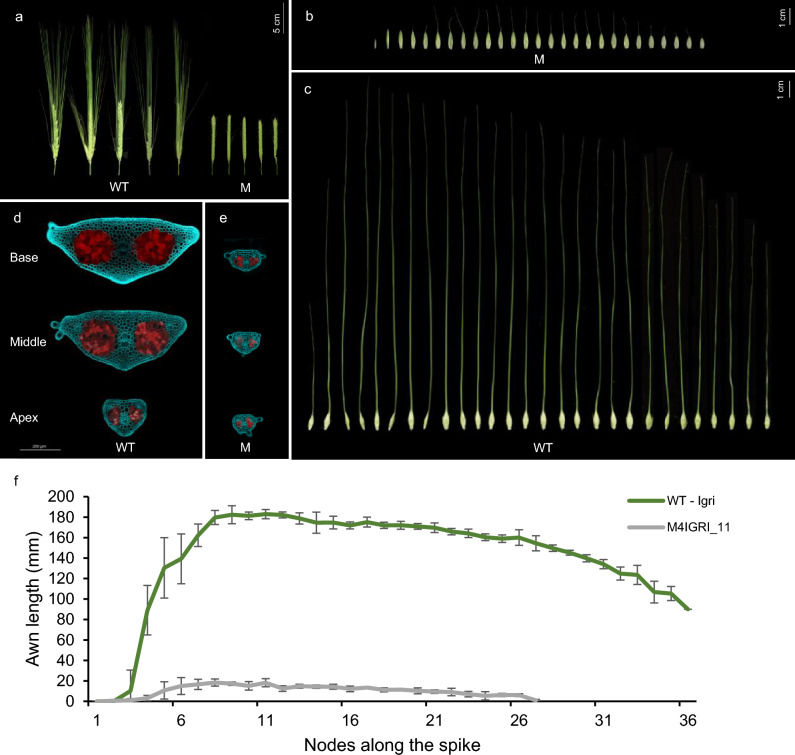
Morphological comparison of the wild-type Igri (WT) and the pendant awn mutant M4IGRI_11 (M). (**a**) Spikes from the primary tiller and spikelets from the primary spike of the (**b**) pendant awn mutant M4IGRI_11 (M), and (**c**) wild-type Igri (WT) at growth stage W9.0. Confocal laser scanning micrographs showing autofluorescence images from 50–100 μm thick cross sections of awns at the base, middle, and apex of the (**d**) WT and the (**e**) M4IGRI_11 plants. Emitted autofluorescence of the lignin-rich cell walls is in cyan (405–510 nm) and chlorophyll in red (660–695 nm) after excitation with a 405 nm laser line. (**f**) Awn lengths corresponding to the nodes along the spikes of WT and M4IGRI_11 plants. The error bars indicate the standard deviation across tested biological replicates (*n* = 5)

The EMS mutant M4IGRI_11 was identified from an M_3_ population in a forward genetics screen based on its distinct brittle, thin and non-erect awns, hereafter referred to as the “pendant awn” phenotype (Fig. 1a–c). In a screen for pleiotropic qualitative morphological effects associated with the underlying mutation, we examined macro- and microscopic differences in the awns and other floral organs. Confocal laser scanning microscopy revealed a uniformly narrow cross sectional surface area in the apical, central and basal parts of the awn in mutants. Unlike the typical tapered apex in WT Igri (Fig. 1d), mutants maintained a uniform and consistently shorter diameter and reduced surface area along their lengths (Fig. 1e). This reduced thickness was a consequence of overall reduction in cell number, rather than cell size. Epidermal and parenchymal cells appeared comparable in size between the WT Igri and the mutant awns, although the precise number and size were not quantified. While mutant awns retained the overall tissue organization, including one central and two lateral vascular bundles and two chlorenchymatous zones, cell number was diminished. The mutants were marked by poor vasculature and weaker cell wall autofluorescence in epidermal and parenchymal cells, suggesting altered cell wall composition and potentially reduced cell wall strength. This possibly compromises mechanical strength, especially at the awn base. Awn length in mutants (10.3 ± 6.5 mm) showed a 93.3% reduction compared to the wild-type (WT) awns (151.9 ± 8.9 mm), making this the most prominent morphological difference (Fig. 1f).

**Fig. 2:**
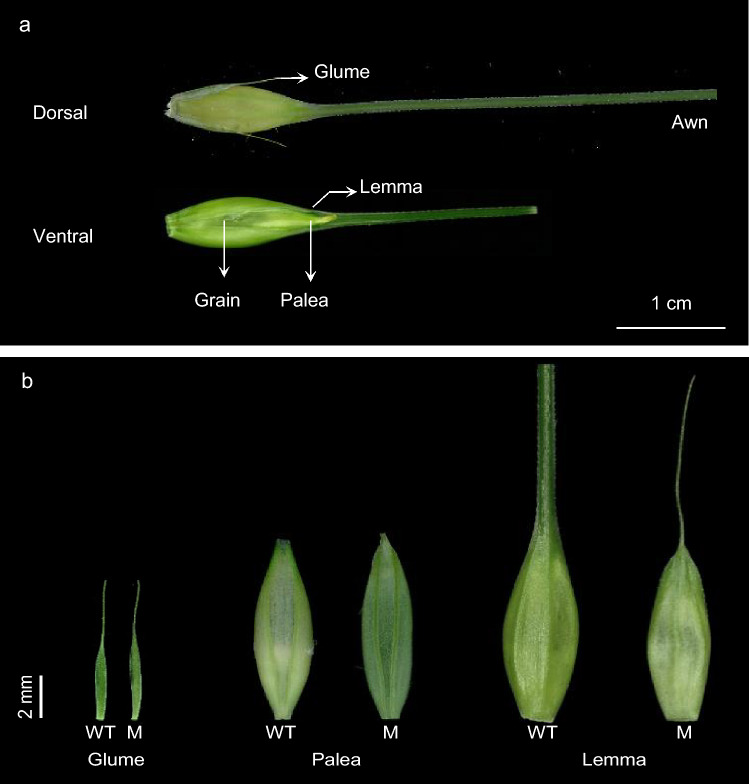
Morphology of the glume, palea and lemma. (**a**) Representative images from the dorsal and ventral sides of a typical barley spikelet from WT Igri. (**b**) Morphology of the glume, palea and lemma from wild-type Igri (WT) and the M4IGRI_11 mutant (M). Spikelets from primary spikes were examined at the time of anthesis (W9.0-W10.0)

Beyond awns, additional morphological differences were detected in whole spikes. Mutant spikes averaged 25.7 nodes/internodes, 16.0% shorter than WT spikes that possessed 30.6 nodes/internodes. While glume size and structure remained unaltered, the mutant palea was narrower and showed reduced curvature compared to WT. Notably, the mutant lemma was significantly shorter and lacked the typical elongation that differentiates into the awn (Fig. 2). Interestingly, the diminished apical extensions in the lemmas of the pendant awn closely resembled the uniformly narrow extensions typically observed in the glume. Transverse sections of palea and lemma from WT and mutants also revealed differences in cellular organization. In WT palea, vascular bundles were surrounded by densely packed parenchyma with well-defined thickened walls, while the mutant palea showed loosely arranged mesophyll cells with less distinct cell layering. (Supplementary Fig. 1). Although not tested statistically, poor grain filling and aborted spikelets at the apex and the base of the mutant spikes were more anecdotal observations (Supplementary Fig. 2a and 2b). Furthermore, mature grains of M4IGRI_11 appeared attenuated and shriveled in comparison with the WT Igri (Supplementary Fig. 2c–f).

An altered flag leaf morphology represented an additional pleiotropic effect of the mutation, in the form of increased flag leaf surface area, length and width in M4IGRI_11 compared to the wild-type (Supplementary Fig. 3).

### Delayed awn outgrowth in M4IGRI_11 spike meristems is evident in early developmental stages

**Fig. 3:**
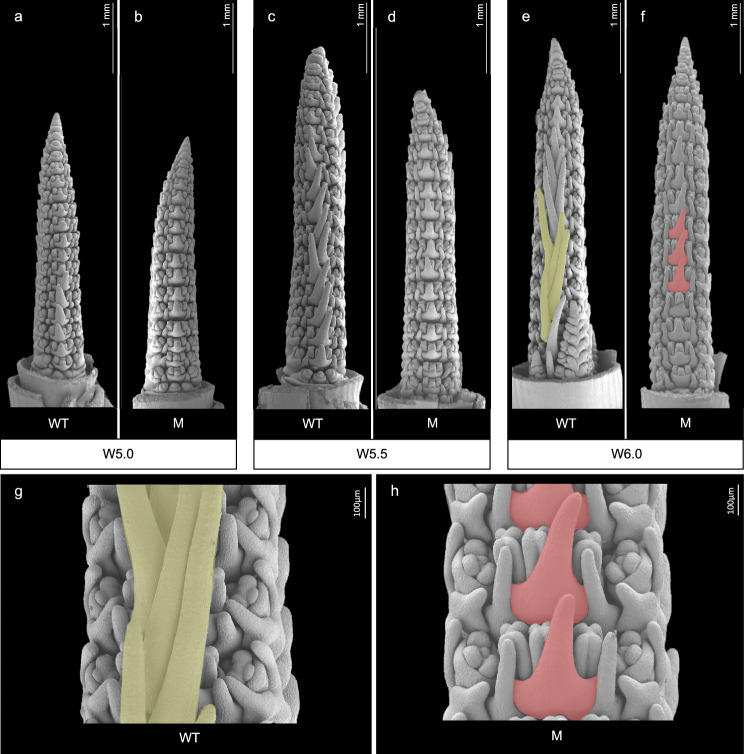
Scanning electron micrographs of developing spike meristems in wild-type Igri (WT) and the M4IGRI_11 mutant (M) across early developmental stages (W5.0, W5.5 and W6.0). Representative images of immature spike meristems dissected from the WT and M4IGRI_11 at successive Waddington stages (**a**, **b**) W5.0, (**c**, **d**) W5.5, and (**e**, **f**) W6.0. White bars = 1 mm. Enlarged view at 100 × magnification from (**g**) WT Igri, and (**h**) M4IGRI_11 spike meristems at Waddington stage W6.0. Yellow and red highlights refer to the magnified areas showing morphological differences in awn primordia across the WT and M4IGRI_11, respectively

To further determine the developmental origin of the observed awn phenotypes, we performed scanning electron microscopy on spike meristems from Waddington stages W4.0–W6.0 comparing awn growth patterns in WT Igri and M4IGRI_11. At earlier stages (W4.0–4.5), no observable differences in organ primordia shape or organization were detected, suggesting conserved meristem identity and indicating that the mutation does not affect basic pattern of ridge initiation or initial reproductive transition (Supplementary Fig. 4). While awn initiation in M4IGRI_11 was comparable to the WT, impaired awn elongation became apparent from stage W4.5 onwards (Fig. 3a–f). By stage W6.0, WT spikes displayed well-defined awn outgrowth zones, indicative of active elongation of lemma-awn structures (Fig. 3g), while awn growth in the mutant was visibly suppressed (Fig. 3h). These findings suggest that although early meristem identity and reproductive transition remain phenotypically unaffected, the mutation disrupts developmental progression at or after awn founder cell differentiation, ultimately leading to the mature pendant awn phenotype in M4IGRI_11.

### A single recessive gene mapped on chromosome 3H controls the pendant awn phenotype

To understand the genetic basis of the pendant awn phenotype in M4IGRI_11, we generated and phenotyped an F_2_ population originating from a cross between the M_5_ pendant awn mutant M4IGRI_11_11 and the two-rowed winter barley cultivar ‘Alraune’. Among 149 phenotyped plants, twenty-eight F_2_ plants (18.8%) showed the mutant trait. The ratio of WT to mutant plants supported a monogenic, dominant-recessive segregation pattern, indicating a single-locus control of the phenotype (*χ*^2^ = 3.06, df = 1, *χ*^2^_0_․_95;1_ = 5.02, two-sided; Supplementary Table 1).

**Fig. 4:**
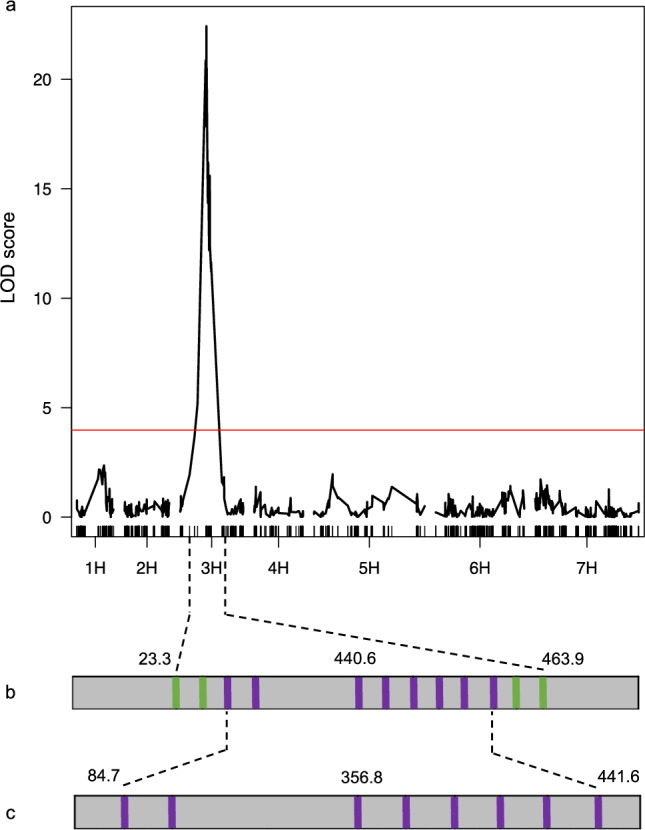
Mapping the pendant awn phenotype to chromosome 3H in barley. (**a**) Genome-wide linkage analysis of 1,128 GBS-based SNP markers across all seven barley chromosomes (1H-7H) using a segregating F₂ population (*n* = 149) from a cross between M4IGRI_11_11 and Alraune. Vertical black bars represent the position of individual markers along the genetic map in centimorgans (cM). Logarithm of odds (LOD) scores are plotted against genetic positions, with the red horizontal line indicating the significance threshold determined by 1,000 permutation tests. Narrowing down of the target interval using (**b**) GBS- and KASP-based SNP markers and (**c**) graphical genotyping using KASP markers on F_3_ progeny originating from 24 selected F_2_ parents with recombination events within the interval to a final window of 356.8 Mbp on chromosome 3H. Green and purple bars represent the physical positions of GBS-based and KASP markers, respectively, in Megabase pairs (Mbp), with numerical coordinates shown above the flanking markers. Numbers between dotted intervals are the physical distances encompassed in Mbp

For genetic mapping, we performed Genotyping-by-Sequencing (GBS) on the same F_2_ panel, including parental controls (WT Alraune and Igri, and M4IGRI_11_11). On average, 3.3 million reads per sample were generated with a mapping rate of 71.4% against the Igri reference genome (Jayakodi et al. 2024). After variant calling and filtration for mapping quality, read and allele depth, missingness and minor allele frequency, 1,128 high-confidence single nucleotide polymorphisms (SNPs) were retained (Supplementary Fig. 5, Supplementary Table 2). A genome-wide QTL scan assuming a single locus for the pendant awn trait revealed twenty-eight SNP markers significantly associated (LOD > 4.1, permutation test, *n* = 1,000) with the phenotype on chromosome 3H (Fig. 4a), spanning 31.1 cM corresponding to a physical interval of 440.6 Mbp (Fig. 4b). Using the GBS dataset, we analyzed graphical genotypes within this 440.6 Mbp interval on chromosome 3H in 146 F_2_ individuals (Supplementary Fig. 6a). A subset of 29 recombinants showing crossovers from homozygous mutant to heterozygous or homozygous WT alleles was selected to refine the locus (Supplementary Fig. 6b). We generated F_3_ families from these recombinants since homozygous dominant and heterozygous genotypes were phenotypically indistinguishable in F_2_ population. The pendant awn phenotype consistently co-segregated with homozygous mutant alleles, whereas WT individuals were either heterozygous or homozygous for the Alraune allele, thereby confirming the genotype–phenotype relationship.

For the equidistant physical saturation of the 31.1 cM (440.6 Mbp) target interval (Fig. 4b), 15 polymorphic KASP markers (1/31 Mbp) were designed using the GBS dataset. Ten of these markers proved polymorphic and were used for fine-mapping. Validation of F_2_ genotypes through F_3_ family genotyping revealed discrepancies in five families, likely due to mix-ups during tissue sampling and/or DNA isolation. These included unexpected heterozygous calls at markers that were homozygous in the F_2_ parents, leading to exclusion of these families (Supplementary Table 3). We then analyzed graphical genotypes from 203 F_3_ individuals (derived from 24 F_2_ recombinants) using 9 KASP markers (Supplementary Fig. 7) within the previously defined 440.6 Mbp region, which reduced the candidate interval to 356.8 Mbp (Fig. 4c).

### *EMBRYONIC FLOWER 1 LIKE (EMF1L)* is likely the gene responsible for the pendant awn phenotype barley

Since the pendant awn phenotype resulted from EMS mutagenesis, mutants and WT plants should differ by the presence or absence of EMS-specific variants within the mapped 356.8 Mbp interval on chromosome 3H. Given the large size of the interval spanning 57.3% of chromosome 3H, we performed whole-genome re-sequencing (WGS) to identify mutant-specific variants for the discovery of the candidate gene(s) within the interval. We sequenced M4IGRI_11_11, five independent mutants from the same Igri-EMS population exhibiting unrelated phenotypic changes, and WT Alraune, generating three WGS libraries for each. De-duplicated uniquely mapped reads accounted for an effective coverage of 6.6x ± 1.0 × along the Igri reference genome (4.2 Gbp) (Supplementary Table 4) with a mapping rate of 90.7% ± 1.1%. We identified 60,329 variants post-filtration in M4IGRI_11_11. Variants shared between the WT Alraune and M4IGRI_11_11 were excluded, resulting in 9,996 genome-wide variants exclusive to the pendant awn mutant (Supplementary Table 5). Of these, 4,232 (42.3%) were C to T and G to A transitions consistent with the canonical EMS mutation pattern. We also observed 7.7% non-canonical transitions, 32.1% transversions and 17.7% insertions or deletions (InDels), with insertions up to 24 bp and deletions up to 16 bp.

To predict polypeptide-level variant effects, we examined 955 variants-852 single nucleotide variants (SNVs) and 103 InDels unique to the M4IGRI_11_11 pendant awn mutant within the 356.8 Mbp target interval on chromosome 3H. This region contained 2,257 annotated genes, 19 of which harbored SNVs with predicted effects within exons or introns. These included premature stop codon gained, missense, synonymous and intronic variants (Supplementary Table 6). The majority (97.8%) of variants were located in intergenic regions or within 5 Kb up- or downstream of genes relative to their translation start and stop sites, respectively, with 34 genes associated with the latter. While regulatory effects from such variants cannot be ruled out, for candidate gene selection, we prioritized variants within the coding sequence (CDS) with SNVs predicted to have high-impact consequences on protein function. This applied to three annotated genes: *HORVU.IGRI.PROJ.3HG00221700.1* affected by a premature stop codon and *HORVU.IGRI.PROJ.3HG00723070.1* and *HORVU.IGRI.PROJ.3HG00234510.1*, each carrying a missense mutation. The premature stop codon mutation caused by an A to T SNV was predicted to truncate the protein after 471 amino acids, resulting in a 62.3% loss of the full-length polypeptide. In the barley pan-genome version 2 (BPGv2) database- https://panbarlex.ipk-gatersleben.de/ (Jayakodi et al. 2024), *HORVU.IGRI.PROJ.3HG00221700.1* was a high-confidence gene functionally annotated as *EMBRYONIC FLOWER 1 LIKE (HvEMF1L)*. The other candidate genes carrying missense mutations, *HORVU.IGRI.PROJ.3HG00723070.1* and *HORVU.IGRI.PROJ.3HG00234510.1* were either low confidence genes and likely fragmented and mis-annotated in the BPGv2 or showed no detectable expression levels when screened using the barley pan-transcriptome dataset (Guo et al. 2025), respectively. Thus, *HvEMF1L* was prioritized as the strongest candidate underlying the pendant awn trait.

### *HvEMF1L* is highly conserved across global barley germplasm and significantly upregulated in the inflorescence

**Fig. 5:**
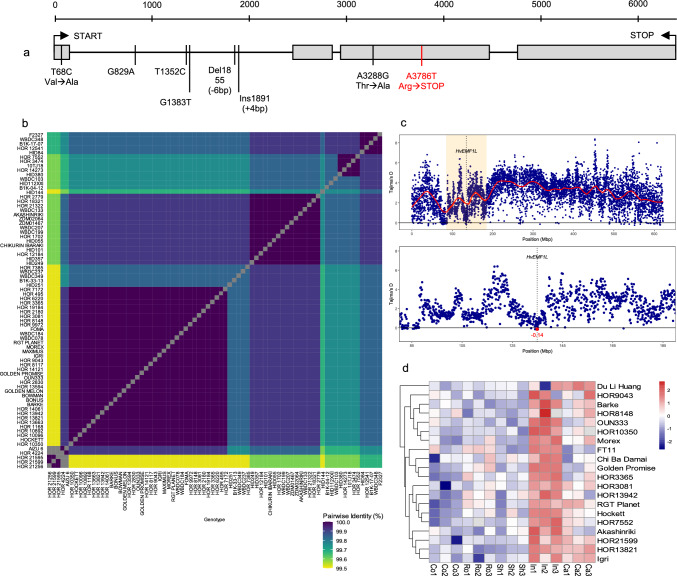
Genomic and transcriptomic features of *HvEMF1L* across global barley germplasm. (**a**) Gene model of *HvEMF1L* showing the exon–intron structure based on the Igri reference genome annotation and physical positions of natural variants (labeled in black) and their consequences identified in 1,000 genebank accessions (landraces and early cultivars) and 315 barley elite cultivars. The EMS-induced mutation characterized in this study is highlighted in red. START and STOP indicate the translation start and termination codons, respectively. Numbers refer to the physical position of the variants relative to the START site. (**b**) Pairwise identity matrix based on HvEMF1L polypeptide sequence alignments from the 76 BPGv2 genotypes (Jayakodi et al. 2024), illustrating high sequence conservation in the panel. Gray boxes represent 100% sequence identity. (**c**) Tajima’s D along chromosome 3H. Genome-wide distribution of Tajima’s D in 100 kb windows (top), with smoothened curve (red). The orange rectangle marks the ± 50 Mbp region surrounding *HvEMF1L* (dotted black line at midpoint). Zoomed-in view of the highlighted region (bottom). The red point indicates the Tajima’s D value from the bin harboring *HvEMF1L*. (**d**) Heatmap of *HvEMF1L* expression profiles from the barley pan-transcriptome dataset (Guo et al. 2025) of 20 barley genotypes across five tissues: embryo (Co), root (Ro), shoot (Sh), inflorescence (In), and caryopsis (Ca) with three biological replicates each (suffixed with 1, 2 and 3). Color scales between blue (low) and red (high) represent expression profiles as normalized Log_2_ fold change values

Due to the high plasticity of awn morphology in barley natural diversity collections (Milner et al. 2019) we questioned the potential functional relevance and natural variation in *HvEMF1L*. Therefore, we analyzed genomic and polypeptide sequences of the gene from the 76 whole-genome assemblies of the BPGv2 (Jayakodi et al. 2024) and from re-sequencing data of the 1,315 diverse barley genotypes (Milner et al. 2019). Across all genotypes, seven high-confidence natural variants were detected, comprising five single nucleotide polymorphisms (SNPs) and two insertion–deletion events (−6 bp and + 4 bp; Fig. 5a). Of these, only two SNPs were located within the CDS: T68C, which results in a conservative amino acid substitution from Valine to Alanine, and A913G, which causes a Threonine to Alanine change. Within the BPGv2 genotypes, *HvEMF1L* was present as a single copy gene in all 76 genotypes. Moreover, multiple sequence alignments of *HvEMF1L* genomic sequences revealed 99.9% pairwise identity with 99.6% identical sites (Fig. 5b), suggesting that it is highly conserved and likely an essential gene intolerant of mutations. This was further supported by chromosome-wide estimates of Tajima’s D across chromosome 3H that revealed broad variation in allele frequencies (Fig. 5c). Around the *HvEMF1L* locus (134.89–134.90 Mbp), a distinct local pattern was detected. Flanking bins showed increased Tajima’s D values, while the window overlapping the gene midpoint dipped to -0.14, suggesting that diversity appears to accumulate around but reduced within the gene itself.

Using the barley pan-transcriptome dataset from 20 diverse barley genotypes (Guo et al. 2025), we examined the expression profile of *HvEMF1L* across the five sampled tissues—embryonic, root, shoot, inflorescence and caryopsis (Fig. 5d). *HvEMF1L* was significantly upregulated in the inflorescence in most genotypes, with mean log-expression differences of 0.61 compared to root, 0.72 to shoot, 0.79 to embryo, and 0.43 to caryopsis (all *p* < 0.001).

## Discussion

Understanding the genetic control of awn development is important, since awns contribute to photosynthesis, transpiration, carbohydrate accumulation and drought stress mitigation, potentially maintaining grain yield under stress in cultivated grasses (Guo and Schnurbusch 2016; Ntakirutimana and Xie 2019; Sanchez-Bragado et al. 2023) and are an essential part of the seed dispersal and burial system in non-domesticated crop wild relatives (Elbaum et al. 2007; Petersen and Kellogg 2022). Additionally, awns serve as a useful morphological proxy for studying leaf development (Patterson et al. 2025; Zhang et al. 2024a). To investigate the genetic control of awn development, we leveraged EMS mutagenesis as a forward genetics tool and identified the pendant awn mutant, M4IGRI_11, characterized by thin, brittle and short awns. The pendant awn trait follows Mendelian segregation and is monogenically inherited. By combining whole-genome re-sequencing with additional mutant analyses, we resolved the limitations of low-resolution mapping to localize the underlying gene. Using a multi-step mapping approach, we identified a mutant-specific SNP in the *EMBRYONIC FLOWER 1 LIKE (HvEMF1L*) gene, introducing a premature stop codon that truncates 62.3% of the encoded protein likely resulting in loss of function. Although this gene was recently implicated in barley awn development via the *short and crooked awn* (*sca*) mutant (Nakamura et al. 2025), our findings independently reinforce its role in a different genetic background with a distinct mutant allele.

Our study noted significant morphological defects in the pendant awn mutant compared to the wild-type (WT) Igri. The pendant awn mutant showed pleiotropic spike defects, marked by reduced lemma and palea size, initially thought to result from changes in cell number and size or missing specific cell types altogether. Microscope-based qualitative analyses showed reduced parenchymal cell counts in the awns, with largely conserved cell sizes, suggesting a primary defect in cell division rather than expansion. Since inflorescence development involves sequential phases of cell division followed by expansion (Krizek and Anderson 2013), impaired cell proliferation potentially due to mis-regulation of cyclins, CDKs, or hormonal crosstalk (Tank and Thaker 2011) may underline the phenotype. Several genes are known to modulate cell division in cereal awns, including *an-1* and *gla1* in rice (Luo et al. 2013), *ali-1* in wheat (Wang et al. 2020) and *lks2* in barley (Yuo et al. 2012). While these genes mainly influence longitudinal cell number, cross sectional cell counts are maintained. In contrast, the pendant awn mutant displayed reductions across both dimensions, further suggesting a broader defect in cell division. Therefore, quantitative analyses are still required to validate cell size consistency and the magnitude of cell number reduction in the mutants. Mutant awns were not uniformly upright due to structural instability that might be linked to cell numbers and the cell wall composition. Fainter autofluorescence signals in cell walls of basal awn segments are indicative of reduced lignin content, which when combined with reduced cellulose and hemicellulose content may compromise structural integrity and reduce tissue rigidity (Kokubo et al. 1991; Nakamura et al. 2025).

As an important source tissue, flag leaves contribute significantly to the grain-filling process and therefore affect grain yield (Xu et al. 2024). Since they differentiate from the vegetative meristem as early as stage W1.5 prior to floral transition (Vahamidis et al. 2014), the observed flag leaf enlargement in the pendant awn mutants may suggest a pleiotropic effect of the gene on both vegetative and reproductive development. In spike meristems, morphological divergence was apparent through delayed or loss in lemma and awn initiation after stage W4.5. Homeotic transformation of floral bracts has been reported in cereals. For example, in common wheat, the domestication allele *Q*, encoding the *APETALA2* (*AP2*) transcription factor, promotes the development of soft glumes; previously of unclear origin but later identified as lemma-like structures or sterile lemmas (Song et al. 2019). Similarly, in rice, *LONG STERILE LEMMA1* (*G1*) represses lemma identity to maintain the sterile lemma (Liu et al. 2016; Yoshida et al. 2009). While these examples highlight the plasticity of floral bract identity in grasses, they also support our hypothesis that *HvEMF1L* may function to preserve lemma identity by repressing a glume-like fate. Loss of *HvEMF1L* function may result in the homeotic conversion of lemmas to glume-like structures, indirectly suppressing awn initiation. This was evident in our macroscopic observations, where the uniformly narrow apical extensions of WT glumes resembled the diminished extensions of the mutant lemmas. Studies have investigated barley grain size and weight as a function of lemma and palea growth since they protect the inner floral organs and ensure stable growth, seed setting and grain filling (Zhang et al. 2024a). Although not statistically validated, anecdotal comparisons in grain size between mutants and WTs suggest that reduced photosynthetic input from the attenuated paleae, poorly vascularized lemmas and shortened awns may have an impact on grain development. Further quantitative analyses are required to determine whether the pleiotropic effects of the pendant awn mutation also extend to grain yield.

Interestingly, *HvEMF1L* was functionally annotated as the *EMBRYONIC FLOWER 1 LIKE* gene, but shares only 15.6% and 39.4% pairwise polypeptide sequence identity with its putative orthologs in Arabidopsis (Aubert et al. 2001; Shu et al. 2024) and rice (Yan et al. 2015), respectively. Diversity analyses of HvEMF1L across global barley germplasm (Jayakodi et al. 2024; Milner et al. 2019) revealed high conservation among wild accessions, landraces and elite cultivars, with narrow genetic diversity and very few coding-region polymorphisms. A negative Tajima’s D value can be associated with purifying selection removing deleterious variants or with recent selective sweeps. Since the values were close to neutrality at −0.14, demographic processes such as expansion or local recombination cannot be truly ruled out. Nevertheless, the analyses reveal a pattern for *HvEMF1L* that is consistent with functional constraint via resisting the accumulation of polymorphisms. The pan-transcriptome analyses identified an inflorescence-specific upregulation irrespective of origin or growth type. Such spatial specificity in expression reinforces its putative involvement in regulating inflorescence architecture, possibly through maintaining floral organ identity and coordinating cell differentiation within the spikelet. Furthermore, the rarity of coding SNPs and high sequence identity position *HvEMF1L* as a core gene in inflorescence development via cellular regulation. Given its weak expression in vegetative tissues, *HvEMF1L* likely acts downstream or independently of general developmental regulators and instead responds to floral-specific cues. Future studies could aim to functionally characterize *HvEMF1L* and elucidate its co-regulatory gene modules in the context of floral initiation. While *HvEMF1L* is considered as the strongest candidate, functional validation through targeted mutagenesis are needed to confirm causality.

## Materials and methods

### Plant material and growth conditions

The M4IGRI_11 mutant was identified from an ethyl methanesulfonate (EMS)-mutagenized population generated using the two-rowed winter-type cultivar ‘Igri’ as the donor. EMS treatment was performed using the protocol of Gottwald et al. (2009). Resulting M_1_ plants were successively self-fertilized to yield segregating M_2_, M_3_ and M_4_ families. The pendant awn mutant was observed at the time of flowering in the M_3_ generation and was selfed to yield the M_4_ mutant—M4IGRI_11. This was further selfed to produce the M_5_ individual M4IGRI_11_11. For morphological and histological analyses, four batches of ten seeds each from wild-type (WT) Igri and M4IGRI_11 were sown weekly. An F_2_ population (*n* = 180 individuals) from selfed F_1_ hybrids of the pendant awn mutant M4IGRI_11 and the two-rowed winter barley cultivar ‘Alraune’, was used for inheritance and mapping studies. As parental controls, WT Igri and Alraune and M4IGRI_11 were grown. Five additional independent mutants from the same Igri-EMS population, exhibiting unrelated phenotypes were used for whole-genome re-sequencing (Supplementary Table 7).

All plants were grown under greenhouse conditions. Seeds were sown in 96- or 24-well QuickPots (Hermann Meyer, Germany) and grown for two weeks under long-day conditions (16 °C during the day and 14 °C at night; 16 h light). Plants were vernalized for six weeks under short-day conditions (4 °C; 10 h light). After vernalization, plants designated for morphological and histological analyses were transferred to 14 cm pots (Hermann Meyer, Germany) and returned to greenhouse conditions. Plants for Genotyping-by-Sequencing (GBS) analyses were kept in the 96-well QuickPots and grown to maturity in a speed breeding cabin (22 °C during the day and 20 °C at night; 23 h light).

### Morphological and histological assessment

Spikes and spikelets from only the primary tillers of M4IGRI_11 and WT Igri were used for macroscopic analyses. Growth stages were determined according to Kirby and Appleyard (1981) and Waddington et al. (1983). Flag leaves, spikes and spikelets were imaged using a Canon Canoscan 8800F flat-bed scanner (Canon Deutschland GmbH, Germany) with resolutions of 300–1200 dpi. Internode counts, and the awn lengths were measured from five spikes per genotype. Flag leaf length, width, and area were measured from M4IGRI_11 (n = 8) and WT Igri (n = 11) plants using the Fiji distribution from ImageJ (Schindelin et al. 2012). Statistical significance was tested with the two-sided independent sample t-test and the Welch’s t-test (α = 0.05) for equal and unequal sample sizes, respectively.

Glumes, lemmas, and paleas from the spikes used in macroscopic analyses were examined under the Keyence VHX5000 digital microscope (Keyence GmbH, Germany). Awn cross sections (apical, central and basal) from M4IGRI_11 and WT Igri plants were examined using an inverted Zeiss LSM780 (Carl Zeiss Microscopy GmbH, Germany) confocal laser scanning microscope. Freshly harvested awns were cut into 1 cm pieces and embedded in 8% agarose and sectioned (50–100 μm thickness) using the Leica VT1000 S vibratome (Leica Biosystems Nussloch GmbH, Germany). For pendant awns, the agarose-embedded samples were transversely sectioned and mounted on Lab-Tec II chamber slides (Life Technologies GmbH, Germany). Sections were imaged using a 405 nm laser line, detecting autofluorescence at 405–510 nm for lignin-rich cell walls and 660–695 nm for chlorophyll (Donaldson 2020). Images were captured using a standard 20 × objective (numerical aperture = 0.8, pinhole diameter = 46 μm, zoom setting = 1.0 and image resolution = 2028 × 2048 pixels).

Early spike development (W2.75-W6.0) in M4IGRI_11 and WT Igri was analyzed using a Zeiss Gemini300 scanning electron microscope (Carl Zeiss Microscopy GmbH, Germany; 5 kV acceleration voltage). Samples were fixed in 4% formaldehyde in 50 mM phosphate buffer (pH 7.2) with 0.05% Triton X-100 for 16 h at 8 °C, then washed twice with distilled water, and step-wise dehydrated with 30, 50, 70 and 90% and finally twice with 100% ethanol. Samples were critical-point dried in a Bal-Tec Critical Point Dryer (Bal-Tec AG, Switzerland) and transferred onto carbon-coated aluminum specimen mounts and coated with gold in an Edwards S150B sputter coater (Edwards High Vacuum Inc., United Kingdom).

### Determining the genetic inheritance pattern

Mature F_2_ spikes were phenotyped in a binary manner with mutant and WT phenotypes referenced against the parental controls Alraune and M4IGRI_11. A Pearson’s *χ*^2^-test was conducted to assess expected and observed frequencies assuming a 3:1 (WT:mutant) segregation ratio under the null hypothesis of a single monogenic dominant-recessive inheritance pattern.

### DNA isolation

Genomic DNA (gDNA) of the F_2_ and F_3_ individuals, and the parental control genotypes Alraune, Igri, and M4IGRI_11 was isolated from leaves of two-week-old seedlings using a modified GTC-NaCl protocol (Milner et al. 2019) in a 96-well plate format. Frozen leaves were pulverized using the MM 400 mixer-mill (Retsch GmbH, Germany) for 30 s at 30 Hz and suspended in 600 μL of pre-heated (65 °C) GTC extraction buffer (1 M guanidine thiocyanate, 2 M NaCl, 30 mM sodium acetate pH = 6.0, 0.2% Tween 20). After thoroughly mixing with the mixer-mill (30 s at 30 Hz) and incubation at 65 °C for 30 min, the suspension was centrifuged at 2,550 × *g* for 30 min and the supernatant was transferred to a 96-well EconoSpin plate (Epoch Life Science, USA). DNA binding and washing (50 mM NaCl, 10 mM Tris–HCl pH 8.0, 1 mM EDTA, 70% ethanol) were performed using a vacuum manifold. Final elution (65 °C pre-heated 100 μL TElight buffer; 0.1 mM EDTA, 10 mM Tris–HCl pH 8.0, 10 min room temperature incubation) was done with a centrifugation step at 2,550 × *g*, 10 min for collection into U96 MicroWell Plates (MACHEREY–NAGEL, Germany). Plates were sealed with 96-well Sealing Mats (Thermo Fisher Scientific Inc., USA) and stored at 4 °C or −20 °C for long term storage. For quality control, one μL of gDNA was checked using agarose gel electrophoresis with 2.5, 5, and 12.5 ng/μL lambda DNA (Thermo Fischer Scientific Inc., USA) as reference. Gel image analysis was conducted using UV irradiation in a BioDocAnalyze instrument (Biometra GmbH, Germany).

For whole-genome re-sequencing, 100 mg leaves from two-week old seedlings of selected plant material (Supplementary Table 7) were collected in 1.5 mL Eppendorf tubes, cut into four pieces and flash-frozen in liquid nitrogen. Samples was pulverized with a Model MM 400 Retsch mixer-mill (Retsch GmbH, Germany) at 30 Hz for 1 min and gDNA was isolated using the DNeasy® Plant Mini Kit (QIAGEN, Germany) following the manufacturer’s instructions. DNA quality was verified with agarose gel electrophoresis (1% agarose, 150 V, 30 min) and gel documentation was done as described above.

All gDNA samples were quantified with the Qubit 2.0 Fluorometer (Thermo Fisher Scientific Inc., USA) using the broad-range assay kit (Thermo Fisher Scientific Inc., USA).

#### Genotyping-by-sequencing (GBS)

For genetic mapping of the pendant awn trait, GBS libraries were prepared from 200 ng gDNA of F_2_ individuals and the control genotypes WT Alraune, WT Igri, and M4IGRI_11, digested with *Pst*I and *Msp*I (New England Biolabs, USA) according to the protocol described by Zhang et al. (2024b). The final library was diluted to 620 pM final loading concentration, and sequenced (single read, custom read 1 sequencing primer (Wendler et al. 2014), 122 cycles [read 1], 8 cycles [index read 1] and 8 cycles [index read 2]) on a NovaSeq6000 device using a SP flowcell (Illumina Inc., USA). GBS reads (.fastq format) were mapped to the Igri reference assembly (Jayakodi et al. 2024) using BWA-MEM v0.7.17 (Li 2013). Sequence alignment map (.sam) files were converted and sorted into binary alignment map (.bam) files using SAMtools v1.16.1 with the default parameters (Danecek et al. 2021). Variants were called using BCFtools v1.15.1 (Danecek et al. 2021) and filtered for ≥ 190 reads across all samples, a mapping quality of ≥ 20 and a read depth of ≥ 5 reads per site. All insertions and deletions were removed and only biallelic single nucleotide polymorphisms (SNPs) with a minor allele frequency ≥ 5% and missingness < 76% were retained. The final SNP matrix was compiled in a variant call format (.vcf) file.

#### Genetic linkage map construction for trait mapping

Genetic linkage maps were generated and visualized using the final SNP matrix on R v4.1.1 (R Core Team 2016) using the “QTL” (Broman et al. 2003), “ASMap” (Taylor and Butler 2017), “utl” (Broman et al. 2003) and “ggplot2” (Wickham 2016) packages. The genetic map was constructed based on linkage disequilibrium and trait mapping was performed by calculating the logarithm of the odds (LOD) scores along the seven chromosomes (1H-7H). A permutation test with n = 1,000 replications defined the statistical threshold. Marker positions within the resulting linkage groups were compared to their physical coordinates determined during read mapping to the Igri reference assembly and the LOD-supported confidence interval was identified. This region was delimited using the PCR Allelic Competitive Extension (PACE) Chemistry (3CR Bioscience Ltd., United Kingdom). Fifteen PACE markers (two flanking and 13 distributed) were designed based on polymorphisms between Alraune and M4IGRI_11. Alternative binding sites were checked using BLAST analyses to avoid off-target binding. Two allele-specific forward primers, along with one common reverse primer were designed using the PACE assay design service (3CR Bioscience Ltd., United Kingdom), labeled with HEX (GAAGGTCGGAGTCAACGGATT) and FAM (GAAGGTGACCAAGTTCATGCT) fluorophore tail sequences. The primers were ordered from Metabion (Metabion international AG) (Supplementary Table 8).

KASP assays were performed for the selected F_2_ and, F_3_ families, M4IGRI_11_11, WT Alraune, and WT Igri in a 384-well plate format. Each reaction mixture contained 2.43 μL of 2 × PACE master mix (with passive reference dye), 1 μL DNA, 1.5 μL ultrapure water, and 0.07 μL primer mix (12 μM each allele-specific primer, 30 μM reverse primer. Plates were heat-sealed using optically clear and water-resistant foils (Häberle Labortechnik GmbH & Co. KG, Germany) and pre-read for a baseline value call using a 7900HT Fast Real-Time PCR System (Thermo Fisher Scientific Inc., USA). A touch-down PCR was then performed using a Bioscience Hydrocycler HC-16 (LGV Biosearch Technologies, United Kingdom): 15 min at 94 °C, 10 cycles (94 °C for 20 s, 65 °C for 1 min decreasing 0.8 °C per cycle), then 30 cycles (94 °C for 20 s, annealing and extension at 57 °C for 1 min). Post-PCR, fluorescence was measured after centrifugation for 20 s at 1000 rpm with the Sequence Detection System v2.4 software (Thermo Fisher Scientific Inc., USA).

#### Whole-genome re-sequencing and data analyses

The concentration of quality controlled gDNA samples was adjusted to 50–250 ng in 15 μL nuclease-free water and used for library preparation with the Nextera DNA Flex Library Prep Kit (Illumina Inc., USA) according to the manufacturer’s instructions. Three independent libraries per sample were generated, pooled in an equimolar manner and sequenced to approximately 20-fold coverage using a S2 flow cell (paired-end, 151 cycles [read 1], 10 cycles [index read 1], 10 cycles [index read 2] and 151 cycles [read 2]). Sequencing involved standard protocols and a NovaSeq6000 device (Illumina Inc., USA).

Raw reads (.fastq format) were quality checked with FastQC (http://www.bioinformatics.babraham.ac.uk/projects/fastqc/) and trimmed using the default parameters of fastp (Chen et al. 2018). Reads were mapped to the Igri reference assembly using BWA-MEM v0.7.17 (Li 2013) with default parameters. Mapped reads (.sam format) from the replicates were merged and converted to.bam files, sorted and duplicates were marked using SAMtools v1.16.1(Danecek et al. 2021) with default settings. Using the “mpileup” function in BCFtools v1.15.1 (Danecek et al. 2021) variants were called with default parameters to generate.vcf files and homozygous variants with a mapping quality (MQ) ≥ 30 and read depth (DP) = 3–20 were retained. Only variants unique to the M4IGRI_11_11 pendant awn mutant were retained. Variants conserved between M4IGRI_11_11, Alraune and the other non-pendant awn EMS mutants were excluded from the dataset. Remaining variants were classified as “canonical” transitions (C to T and G to A) characteristic to the EMS-induced pattern, and all other “non-canonical” transitions and transversions and InDels were filtered into independent.vcf files for variant effect prediction.

#### Candidate gene identification

All filtered variants were characterized for predicted polypeptide-level effects using the Ensemble Variant Effect Predictor tool v110.1 (McLaren et al. 2016) with default parameters in offline mode. Variants within the mapped confidence interval for the pendant awn phenotype were prioritized. Coding sequences (CDS) of genes harboring medium to high-impact mutations (frameshifts, splice site, missense and premature stop codon), were extracted and checked for functional annotations. The CDS were queried for BLAST analyses on NCBI.

#### Sequence conservation, and pan-transcriptome analyses

Genotypes from the Core1000 diversity and 315 elite barley cultivar panel (Milner et al. 2019) and 76 genotypes from the barley pan-genome version 2 (BPGv2) (Jayakodi et al. 2024) were used for comparative sequence analyses of *HvEMF1L*. Pairwise sequence identity values were calculated based on multiple sequence alignments of genomic and polypeptide sequences using Clustal Omega 1.2.2 on Geneious Prime version 2023.1.2 (Biomatters Ltd., New Zealand). Neighbor-Joining phylogenetic trees were constructed using the Jukes-Cantor genetic distance model with 1,000 bootstraps replicates. The identity matrix from the 76 BPGv2 genotypes was formatted as a CSV file for further analyses using R (v4.3.0) within RStudio (Allaire 2012). Hierarchical clustering was performed using hclust() (base R) to investigate phylogenetic relationships and improve interpretability of the heatmap. The raw .vcf file from the Core1000 diversity and 315 elite barley cultivar panel was filtered using VCFtools (Danecek et al. 2011) to retain high-quality SNPs from chromosome 3H with a minor allele frequency ≥ 5% and a maximum of 10% missing data per site. Tajima’s D was calculated in 100 kb windows. Tajima’s D values along chromosome 3H were visualized using ggplot2 (Wickham 2016) in R.

Transcripts per million (TPM) matrices for *HvEMF1L* were extracted from the genotype-specific reference transcript dataset (Guo et al. 2025) and log-transformed [log(TPM + 1)] and z-score scaled per gene prior to visualization. To assess significant tissue-specific expression patterns, the log-transformed TPM data were reshaped into long format with melt() (package “reshape2”). A one-way ANOVA was performed with aov() (base R) to test for significance among tissues, followed by Tukey’s Honest Significant Difference (HSD) post hoc test—TukeyHSD() to determine pairwise contrasts. Significance thresholds were set at *p* < 0.05, *p* < 0.01, and *p* < 0.001. A heatmap was generated using the “pheatmap” package (https://github.com/raivokolde/pheatmap) to display expression variation across genotypes.

## Supplementary Information

Below is the link to the electronic supplementary material.

Supplementary file1 (DOCX 15241 kb)

## Data Availability

The datasets generated and analyzed during the current study are available in the European Nucleotide Archive (ENA), accessible under identifiers PRJEB97226 (Genotyping-by-Sequencing data for mapping the pendant awn trait in the F_2_ population) and PRJEB97227 (whole-genome re-sequencing data for mutant-specific variant detection). All scripts used for data analyses and visualization can be provided by the corresponding author upon request.
