# Supplementary material for: A pendant awn phenotype linked to the EMBRYONIC FLOWER 1 LIKE (EMF1L) gene in barley
Source: Theor Appl Genet. 2025 Dec 19;139(1):9. doi: 10.1007/s00122-025-05105-5 (PMC12717203; doi:10.1007/s00122-025-05105-5)
Supplement: Supplementary file 1 — Supplementary file1 (DOCX 15241 kb) [file 122_2025_5105_MOESM1_ESM.docx]

# Supplementary data

## Supplementary Figures


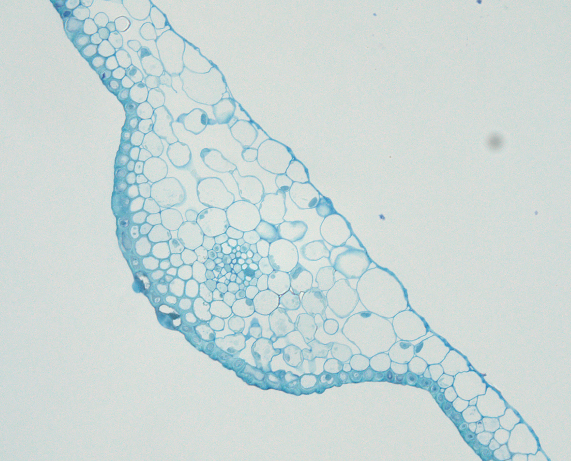

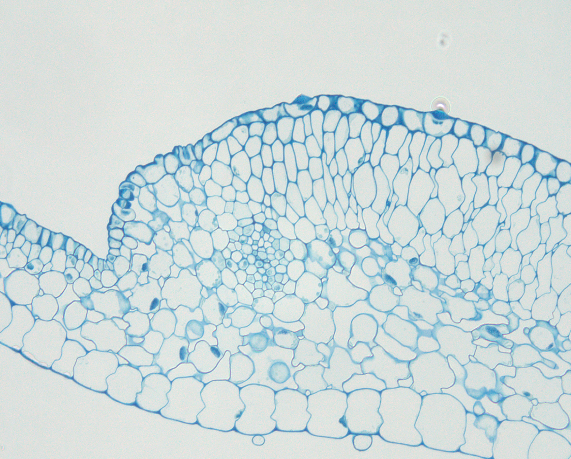


WT

M4IGRI_11

Palea


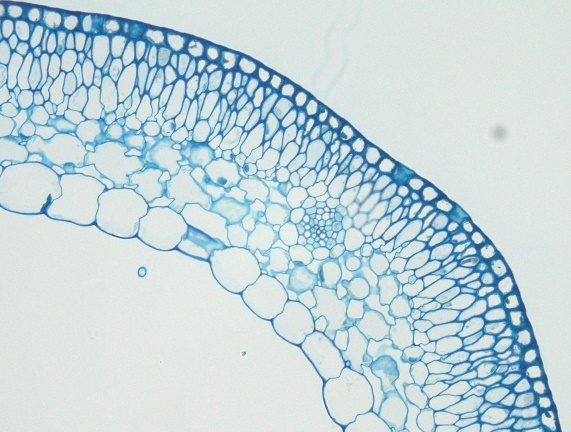

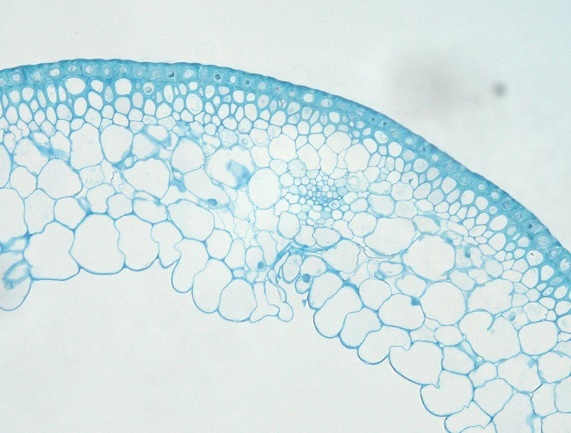


Lemma

Supplementary Fig. 1 Palea and lemma transverse sections of wild-type Igri (WT) and the pendant awn mutant M4IGRI_11 (M). Toluidine blue-stained cross sections of palea and lemma from WT and pendant awn mutants showing vascular bundles surrounded by parenchyma cells. Arrow heads indicate variable cell wall thickenings and cell layering patterns visible across the WT and mutant organs. Images acquired at 40x magnification. Black bar=50 µm.


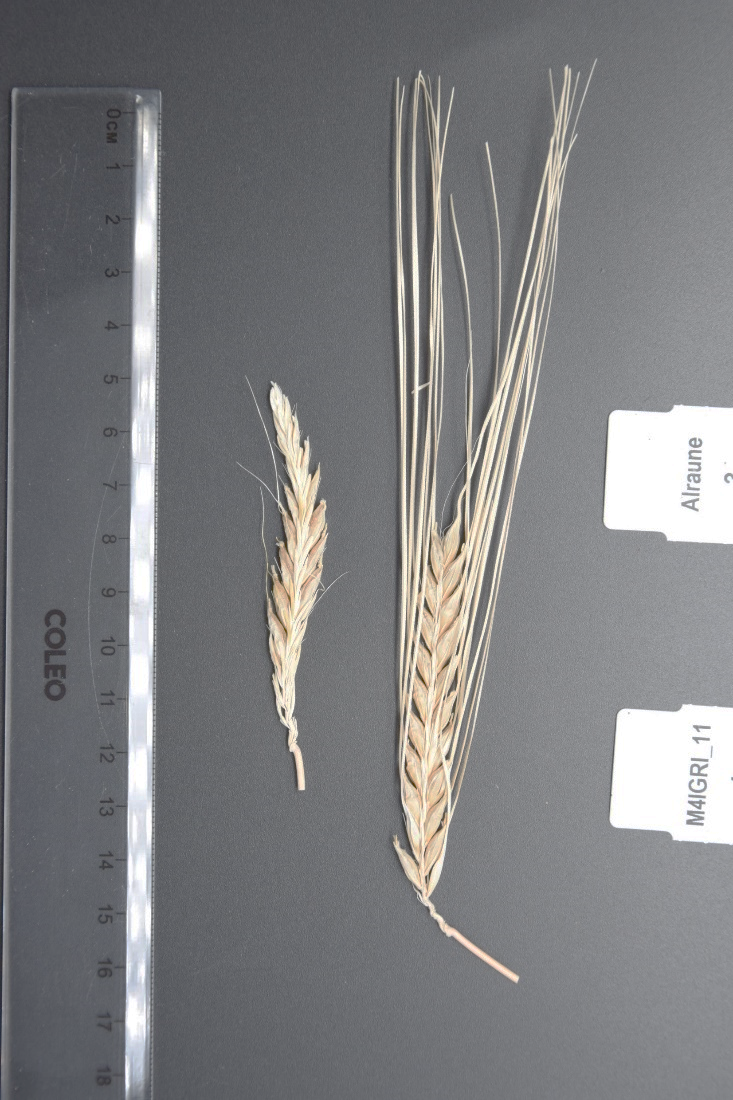

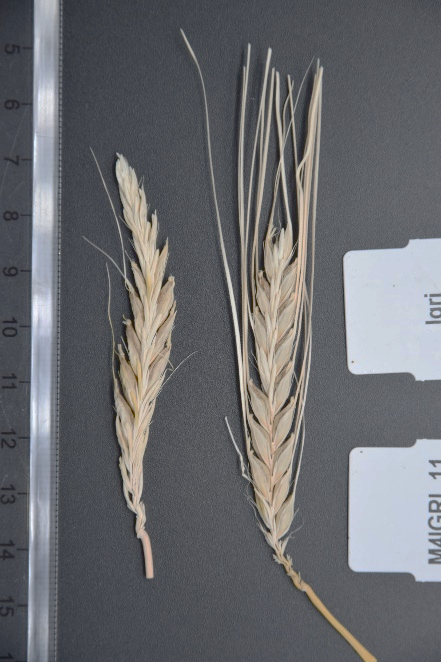

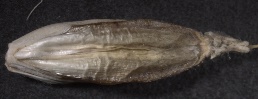

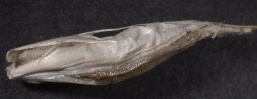

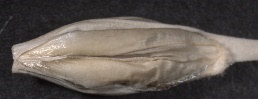

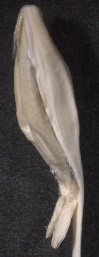


a

b

c

d

e

f

Supplementary Fig. 2 The pendant awn mutation alters spike fertility and grain development. Mature and dried spikes from the primary tillers of the a) M4IGRI_11 mutant (left) and wild-type Alraune (right), b) M4IGRI_11 mutant (left) and wild-type Igri (right). Awns from wild-type spikes were trimmed before image acquisition. Grains harvested from c,d) wild-type Igri and e,f) M4IGRI_11 mutant plants. Yellow bar=1 mm.


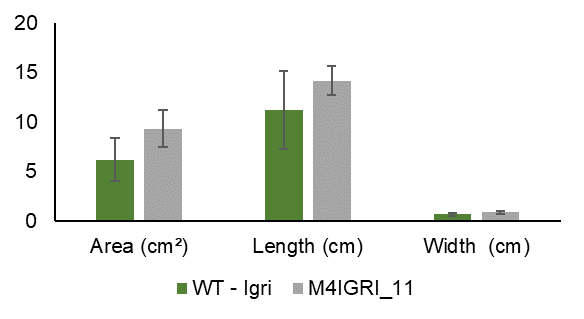


***

***

***

Supplementary Fig. 3 Differences in flag leaf area, length and width between the wild-type Igri and the M4IGRI_11 mutants. Area, length and width of flag leaves of the mutant M4IGRI_11 (gray; n=8) and the wild-type Igri (green; n=11). Asterisks indicate significant statistical differences (Welch’s t-test; *α=*0.05).


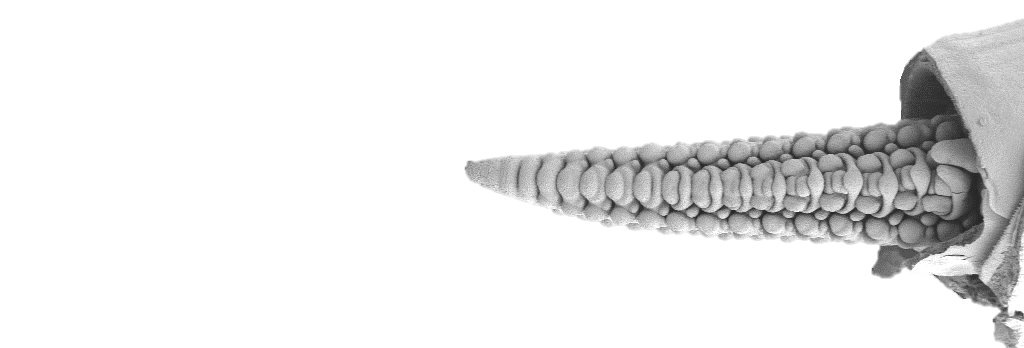

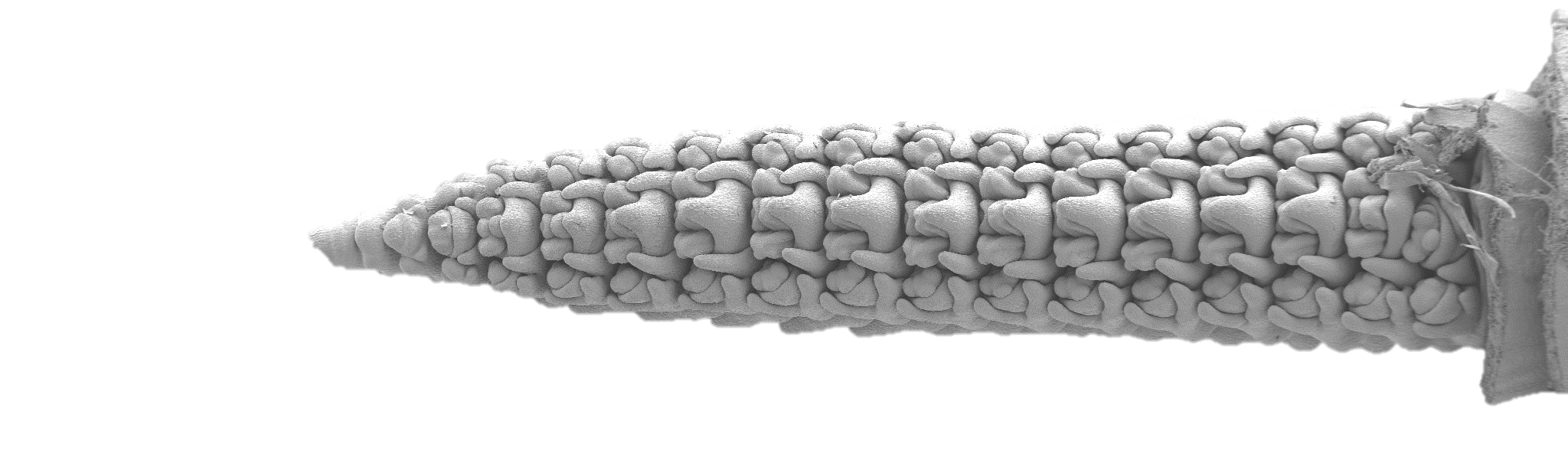


W4.0

WT

M


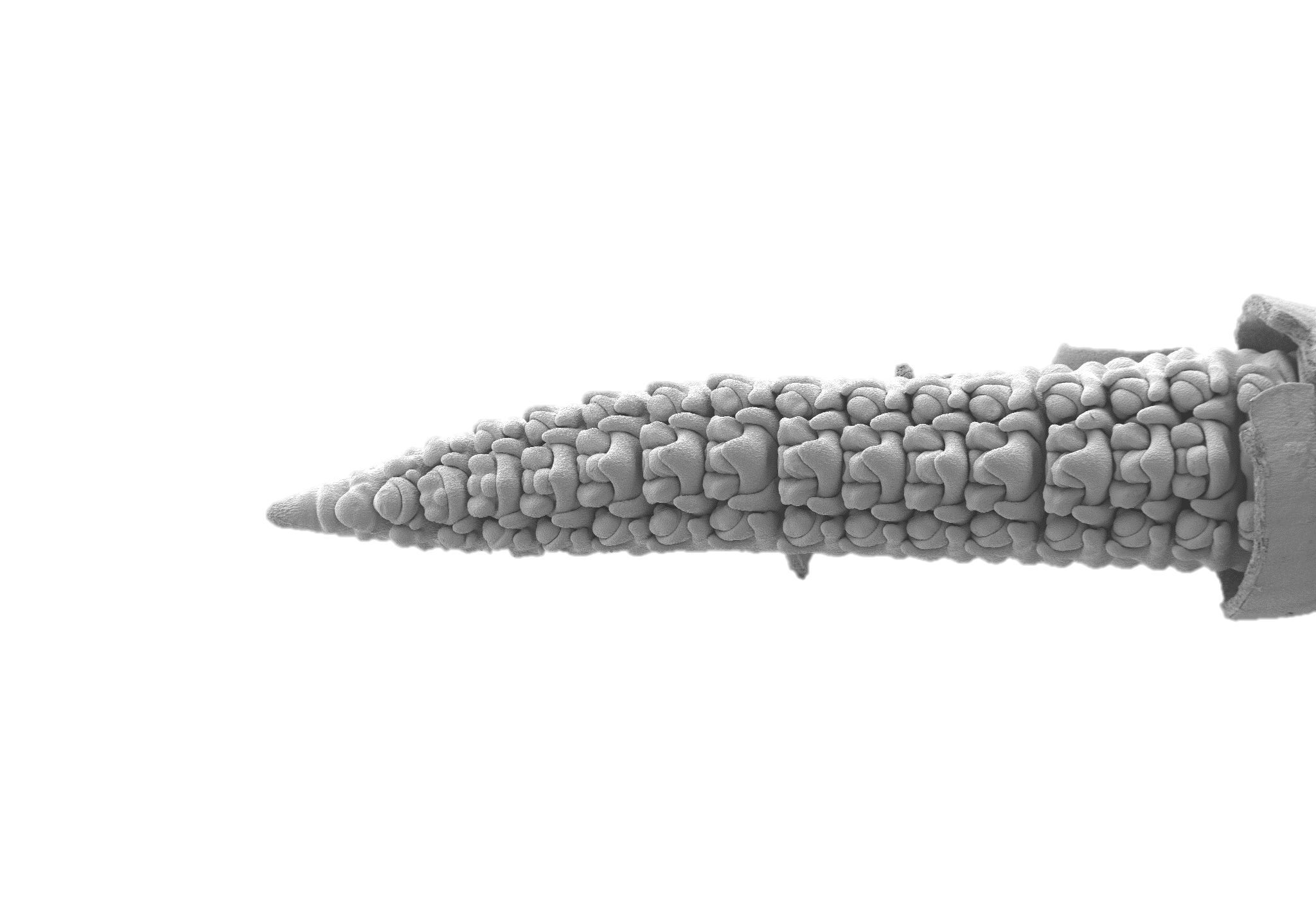

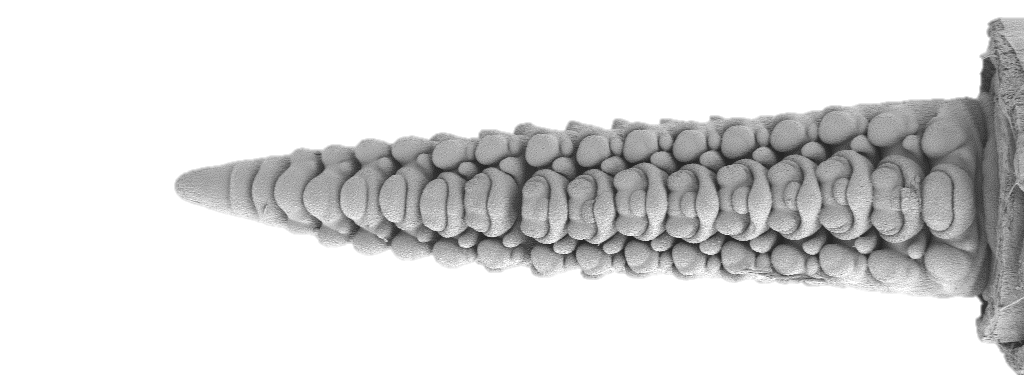


W4.5

WT

M

a

b

c

d

Supplementary Fig. 4 Scanning electron micrographs from immature spike meristems of wild-type Igri (WT) and the M4IGRI_11 pendant awn mutant (M). Representative images of immature spike meristems dissected from the primary tillers of WT and M) at Waddington stages a,b) W4.0 and c,d) W4.5 Spikes were sampled from plants grown under greenhouse conditions (16°C-20°C, long-day photoperiod; 16 h light) with a six-week vernalization period (4°C, 10 h light). White bar=1 mm.


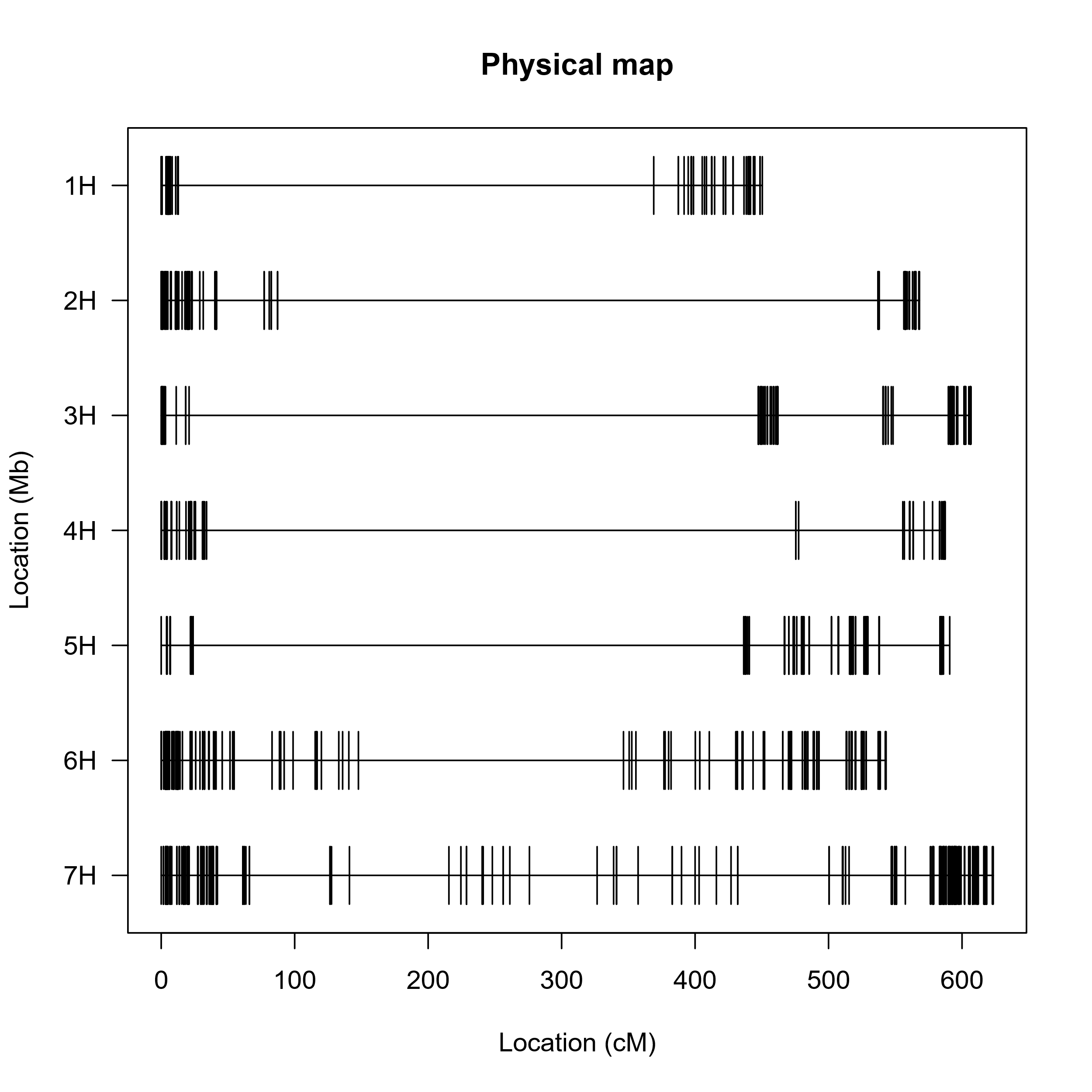


Chromosomes

Physical position (Mbp)

Supplementary Fig. 5 Genome-wide distribution and physical coordinates of 1,128 high-confidence SNP markers used for mapping the pendant awn trait in the F_2_ population. The physical positions in Megabase pairs (Mbp) of the markers along the seven chromosomes (1H-7H) of barley are shown as black vertical lines, based on the Igri reference genome assembly (Jayakodi et al., 2024).

F_2_ individuals

Pendant awn (n=10)

Wild-type (n=19)

23.3

59.4

84.7

103.0

247.7

266.4

285.8

325.8

375.2

441.6

449.8

463.9

365.1 Mbp

b

*AA* (Homozygous wild-type)

*Aa* (Heterozygous)

*aa* (Homozygous mutant)

*AA* (Homozygous wild-type)

*Aa* (Heterozygous)

*aa* (Homozygous mutant)

GBS markers:

KASP markers:

a


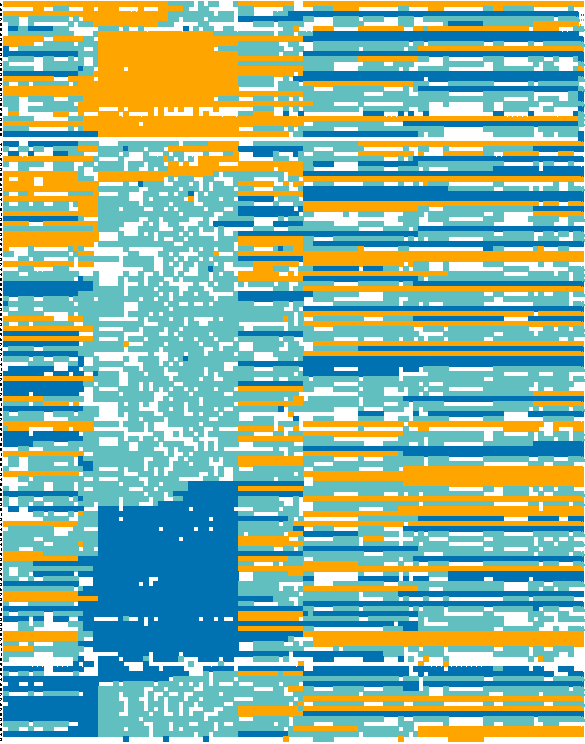


Pendant awn (n=27)

Wild-type (n=119)

🡨Markers along chromosome 3H🡪

F_2_ individuals

440.6 Mbp

23.3

463.9

Supplementary Fig. 6 Graphical genotypes of GBS- and KASP-based SNP markers in the F_2_ mapping population along chromosome 3H. a) Genotypes of 114 GBS-based markers across 146 phenotyped F_2_ individuals derived from the Alraune x M4IGRI_11_11 cross. b) Genotypes of a subset of 29 individuals selected based on presence of recombination events within the 440.6 Mbp interval, using a combination of three GBS based and nine KASP markers. F_2_ individuals are grouped based on their phenotype, with those showing the pendant awn (top) and the wild-type phenotype (bottom). Dark blue and dark green blocks represent homozygous calls for the wild-type Alraune, orange and yellow for the homozygous mutant type, and turquoise blue and light green for heterozygous calls from GBS- and KASP-based markers, respectively. White indicates missing genotyping calls. Vertically oriented black boxes are the intervals which were significantly associated with the pendant awn trait based on linkage mapping. Marker positions are shown as numeric values based on their physical coordinates in Megabase pairs (Mbp) along chromosome 3H as per the Igri reference genome assembly (Jayakodi et al., 2024).

Pendant awn (n=61)

Wild-type (n=142)

F_3_ individuals

356.8 Mbp

*AA* (Homozygous wild-type)

*Aa* (Heterozygous)

*aa* (Homozygous mutant)

KASP markers:

Supplementary Fig. 7 Graphical genotypes KASP-based SNP markers in F_3_ individuals phenotyped for the wild-type and pendant awn trait. Genotypes of 9 KASP-based markers across 203 phenotyped F_3_ progenies derived from 29 F_2_ recombinants within the previously delimited 440.6 Mbp interval. White blocks indicate missing genotyping calls. Marker positions are shown as numeric values based on their physical coordinates in Megabase pairs (Mbp) along chromosome 3H as per the Igri reference genome assembly (Jayakodi et al., 2024).

## Supplementary Tables

Supplementary Table 1 Chi-squared ($\boldsymbol{\chi}$^2^) test evaluating Mendelian monogenic inheritance of the pendant awn trait in the F_2_ population.

| **Phenotype** | **No. F_2_ individuals** | **Observed [%]** | **Expected [%]** | $\boldsymbol{\chi}$**^2*^** |
| --- | --- | --- | --- | --- |
| Pendant awn | 28 | 18.79 | 25 | 2.30 |
| Wild-type | 121 | 81.21 | 75 | 0.77 |
| Total | 149 | 100 | 100 | 3.06 |

*df=1, $\chi_{0.95;1;two-sided}^{2}$=5.025. Observed segregation ratios were tested against the expected 3:1 ratio (wild-type:mutant phenotype) under the hypothesis of a single recessive gene. The table includes the number of individuals scored, observed and expected counts for each phenotype and the $\chi$^2^ values.

Supplementary Table 2 Chromosome-wise counts of GBS-based SNP markers used in the F_2_ mapping population.

| **Chromosome** | **Unfiltered SNPs** | **Filtered SNPs^a^** |
| --- | --- | --- |
| chr1H | 25,498 | 85 |
| chr2H | 32,072 | 130 |
| chr3H | 29,654 | 114 |
| chr4H | 28,079 | 85 |
| chr5H | 29,430 | 115 |
| chr6H | 31,961 | 225 |
| chr7H | 38,440 | 374 |
| chrUn^b^ | 640 | 0 |
| **Total** | 215,774 | 1,128 |

^a^ High-confidence SNP markers with mapping quality ≥ 20, excluding all InDels and including SNP calls with read depth ≥ 5, missingness ≤ 76 % and minor allele frequency ≥ 5 %.

^b^ Unanchored contigs that could not be assigned to any of the seven barley chromosomes (chr1H-chr7H).

Supplementary Table 3 Summary of phenotyping experiments with F_3_ progeny originating from 29 F_2_ individuals recombinant within the target interval.

| **Selected F_2_ parents** | **F_2_ Phenotype** | **No. F_3_ seeds sown** | **No. F_3_ plants left after vernalization** | **No. F_3_ progeny with pendant awns** | **% Pendant awn mutants** |
| --- | --- | --- | --- | --- | --- |
| MZ44-12_2_25 | Mutant | 2 | 1 | 1 | 100.0 |
| MZ44-12_2_48 | Mutant | 2 | 2 | 2 | 100.0 |
| MZ44-12_2_53^a^ | Mutant | 3 | 2 | 2 | 100.0 |
| MZ44-12_2_66 | Mutant | 3 | 1 | 1 | 100.0 |
| MZ44-12_2_69 | Mutant | 13 | 4 | 4 | 100.0 |
| MZ44-12_2_80 | Mutant | 14 | 5 | 5 | 100.0 |
| MZ44-12_2_83 | Mutant | 12 | 6 | 6 | 100.0 |
| MZ44-12_2_143 | Mutant | 2 | 1 | 2 | 100.0 |
| MZ44-12_2_158 | Mutant | 6 | 1 | 1 | 100.0 |
| MZ44-12_2_178^a^ | Mutant | 12 | 5 | 5 | 100.0 |
| MZ44-12_2_1 | Wild | 14 | 8 | All WT | 0.0 |
| MZ44-12_2_8 | Wild | 15 | 7 | 1 | 16.7 |
| MZ44-12_2_11 | Wild | 15 | 8 | 2 | 25.0 |
| MZ44-12_2_20 | Wild | 15 | 10 | 1 | 10.0 |
| MZ44-12_2_31^a^ | Wild | 15 | 9 | 1 | 11.1 |
| MZ44-12_2_35 | Wild | 15 | 13 | 3 | 23.1 |
| MZ44-12_2_42 | Wild | 15 | 14 | 5 | 35.7 |
| MZ44-12_2_54 | Wild | 15 | 11 | 3 | 27.3 |
| MZ44-12_2_55 | Wild | 15 | 6 | 1 | 16.7 |
| MZ44-12_2_63^a^ | Wild | 7 | 5 | All WT | 0.0 |
| MZ44-12_2_95 | Wild | 15 | 9 | 2 | 22.2 |
| MZ44-12_2_99 | Wild | 15 | 12 | 2 | 15.4 |
| MZ44-12_2_109^a^ | Wild | 15 | 11 | 2 | 15.4 |
| MZ44-12_2_113 | Wild | 15 | 14 | 2 | 14.3 |
| MZ44-12_2_144 | Wild | 15 | 10 | 1 | 10.0 |
| MZ44-12_2_148 | Wild | 4 | 3 | 1 | 25.0 |
| MZ44-12_2_156 | Wild | 15 | 4 | 1 | 25.0 |
| MZ44-12_2_167 | Wild | 15 | 7 | All WT | 0.0 |
| MZ44-12_2_174 | Wild | 15 | 10 | 3 | 27.3 |

^a^Removed due to sampling discrepancies.

Supplementary Table 4 Sequencing and mapping statistics from the whole-genome re-sequencing of selected EMS mutants and the wild-type parent controls. Three libraries per individual (indicated by suffixes A-C) were generated and pooled for sequencing.

| **Sample name** | **Raw reads [M]** | **Trimmed reads [M]** | **Trimming rate [%]** | **Duplication rate [%]** | **Coverage without duplicates [x]** | **Mapped reads [%]** |
| --- | --- | --- | --- | --- | --- | --- |
| Alraune-A | 129 | 127 | 1.79 | 57.91 | 5.44 | 88.35 |
| Alraune-B | 134 | 131 | 1.7 | 58.96 | 5.34 | 88.36 |
| Alraune-C | 113 | 111 | 1.66 | 57.04 | 3.54 | 88.38 |
| M3IGRI_3_17_1-A | 574 | 566 | 1.5 | 60.08 | 10.11 | 90.63 |
| M3IGRI_3_17_1-B | 295 | 290 | 1.82 | 56.94 | 5.19 | 91.52 |
| M3IGRI_3_17_1-C | 335 | 331 | 1.41 | 56.4 | 5.91 | 91.36 |
| M4IGRI_4_19-A | 568 | 559 | 1.66 | 59.49 | 5.05 | 90.81 |
| M4IGRI_4_19-B | 287 | 282 | 1.61 | 59.81 | 5.86 | 90.69 |
| M4IGRI_4_10-C | 333 | 328 | 1.65 | 62.48 | 9.99 | 89.97 |
| M4IGRI_8_12_2-A | 622 | 613 | 1.53 | 61.82 | 10.96 | 90.98 |
| M4IGRI_8_12_2-B | 327 | 322 | 1.51 | 60.29 | 5.76 | 91.01 |
| M4IGRI_8_12_2-C | 367 | 362 | 1.43 | 58.42 | 6.47 | 91.25 |
| M4IGRI_10_21_2-A | 332 | 327 | 1.54 | 59.86 | 7.28 | 90.78 |
| M4IGRI_10_21_2-B | 477 | 470 | 1.6 | 58.29 | 3.68 | 91.18 |
| M4IGRI_10_21_2-C | 253 | 249 | 1.57 | 58.61 | 5.85 | 90.69 |
| M4IGRI_11_11-A | 477 | 470 | 1.55 | 62.76 | 8.4 | 90.69 |
| M4IGRI_11_11-B | 253 | 249 | 1.65 | 57.95 | 4.46 | 91.26 |
| M4IGRI_11_11-C | 423 | 417 | 1.42 | 62.36 | 7.45 | 90.99 |
| I-2821-6_2_1-A | 577 | 568 | 1.62 | 57.67 | 10.15 | 91.82 |
| I-2821-6_2_1-B | 314 | 308 | 1.63 | 56.3 | 5.52 | 92.28 |
| I-2821-6_2_1-C | 363 | 357 | 1.61 | 57.22 | 6.39 | 92.05 |

Supplementary Table 5 Summary statistics of high-confidence genome-wide variations unique to M4IGRI_11_11.

| **Variant type** | **chr 1H** | **chr 2H** | **chr 3H** | **chr 4H** | **chr 5H** | **chr 6H** | **chr 7H** | **chr Unn** | **Total** |
| --- | --- | --- | --- | --- | --- | --- | --- | --- | --- |
| C→T^a^ | 526 | 128 | 393 | 42 | 388 | 498 | 470 | 31 | 2,476 |
| G→A^a^ | 443 | 73 | 386 | 75 | 344 | 187 | 223 | 25 | 1,756 |
| Other transitions^b^ | 374 | 24 | 66 | 14 | 54 | 70 | 137 | 37 | 776 |
| Transversions | 816 | 167 | 450 | 106 | 467 | 450 | 595 | 158 | 3,209 |
| Insertions | 94 | 40 | 31 | 31 | 29 | 33 | 39 | 161 | 458 |
| Deletions | 207 | 197 | 163 | 136 | 150 | 143 | 174 | 151 | 1,321 |
|  | 2,460 | 629 | 1,489 | 404 | 1,432 | 1,381 | 1,638 | 563 | 9,996 |

^a^ Canonical C to T/G to A transitions characteristic to EMS-induced mutagenesis.

^b^ All SNPs other than C to T/G to A transitions.

Supplementary Table 6 Effect predictions of single nucleotide variants (SNVs) and insertions and deletions (InDels) within the 356.8 Mbp target interval on chromosome 3H.

|  | **No. variant effects** | |
| --- | --- | --- |
| **Predicted mutation effect** | **SNVs** | **InDels** |
| Stop codon gained | 1 | 0 |
| Missense | 2 | 0 |
| Synonymous | 6 | 0 |
| Intron | 10 | 2 |
| Downstream^a^ | 21 | 7 |
| Upstream^a^ | 14 | 3 |
| Intergenic | 802 | 91 |
| **Total** | 856^b^ | 103 |

^a^ Variants within ±5 Kb of the translation start and stop sites.

^b^ Contains four overlaps.

Supplementary Table 7 Summary of plant lines used for whole-genome re-sequencing.

| **Name** | **Phenotype** | **Generation** |
| --- | --- | --- |
| Igri | Wild-type^a^ | P |
| Alraune | Wild-type^a^ | P |
| M4IGRI_11_11 | Pendant awn | M_5_ |
| I-2821_6_2_1 | Short awned^b^ | M_6_ |
| M3IGRI_3_17_1 | Glume-to-lemma^b^ | M_6_ |
| M4IGRI_4_10 | Leafy lemma^b^ | M_5_ |
| M4IGRI_8_12_2 | Leafy lemma^b^ | M_6_ |
| M4IGRI_10_21_2 | Yellow striped^b^ | M_6_ |

^a^ Wild-type parental controls.

^b^ Non-pendant awn mutants from the same Igri-EMS population used as controls.

Supplementary Table 8 Primer sequences used for KASP genotyping assays targeting SNP markers linked to the pendant awn trait in barley. Each marker is represented by a pair of allele-specific forward primers (Allele-1 and Allele-2) and a shared reverse primer.

| **No.** | **Position (Mbp)** | **Primer** | **Sequence (5'→3')** |
| --- | --- | --- | --- |
| 1 | 187.5 | Allele-1 | GAAGGTGACCAAGTTCATGCTCAATATATGCCCTAAGAGCATCTAAAG |
|  |  | Allele-2 | GAAGGTCGGAGTCAACGGATTAACAATATATGCCCTAAGAGCATCTAAAA |
|  |  | Reverse | CGCGGATGCGTCCGTAGACATT |
| 2 | 594.8 | Allele-1 | GAAGGTGACCAAGTTCATGCTGGAGATATCATGATTCACTTTATAAGTTTC |
|  |  | Allele-2 | GAAGGTCGGAGTCAACGGATTAAGGAGATATCATGATTCACTTTATAAGTTTT |
|  |  | Reverse | CGTGAATTTGAATATTGTGATGAAGTGTTA |
| 3 | 84.7 | Allele-1 | GAAGGTGACCAAGTTCATGCTGATTTTGATTTTATTCTTTTTGCTACGGC |
|  |  | Allele-2 | GAAGGTCGGAGTCAACGGATTGTGATTTTGATTTTATTCTTTTTGCTACGGT |
|  |  | Reverse | TACTCCACAGTAAGATATAATCACTAGGAA |
| 4 | 103 | Allele-1 | GAAGGTGACCAAGTTCATGCTTTCATGTCGGGATTGCTCCCG |
|  |  | Allele-2 | GAAGGTCGGAGTCAACGGATTTCATGTCGGGATTGCTCCCA |
|  |  | Reverse | CTAGTGGTTATGGGGGTGGCTATTT |
| 4^a^ | 103 | Allele-1 | GAAGGTGACCAAGTTCATGCTGTTATGGGGGTGGCTATTTATAGTC |
|  |  | Allele-2 | GAAGGTCGGAGTCAACGGATTGGTTATGGGGGTGGCTATTTATAGTT |
|  |  | Reverse | GGCATTAATGTCATTTCATGTCGGGATT |
| 5 | 156 | Allele-1 | GAAGGTGACCAAGTTCATGCTATGCCCGGTCCGCATCCTC |
|  |  | Allele-2 | GAAGGTCGGAGTCAACGGATTGATGCCCGGTCCGCATCCTT |
|  |  | Reverse | GCAGTTGGTCGCCACCGAGTTA |
| 5^a^ | 156 | Allele-1 | GAAGGTGACCAAGTTCATGCTGTCGCCACCGAGTTAACGACG |
|  |  | Allele-2 | GAAGGTCGGAGTCAACGGATTGTCGCCACCGAGTTAACGACA |
|  |  | Reverse | ACATACGATGCCCGGTCCGCAT |
| 6^a^ | 194.9 | Allele-1 | GAAGGTGACCAAGTTCATGCTGCGCCACCATTTGAGCCATAAC |
|  |  | Allele-2 | GAAGGTCGGAGTCAACGGATTGGCGCCACCATTTGAGCCATAAT |
|  |  | Reverse | GCTATGGTACTAGCGGTGGCCAT |
| 6 | 194.9 | Allele-1 | GAAGGTGACCAAGTTCATGCTGCCATGGCCCTAGCAGCTG |
|  |  | Allele-2 | GAAGGTCGGAGTCAACGGATTGGCCATGGCCCTAGCAGCTA |
|  |  | Reverse | ATAGGCGCCACCATTTGAGCCATAA |
| 7 | 223.3 | Allele-1 | GAAGGTGACCAAGTTCATGCTCAAGTTCCGCCTCGTGAC |
|  |  | Allele-2 | GAAGGTCGGAGTCAACGGATTGCTCAAGTTCCGCCTCGTGAT |
|  |  | Reverse | AAAATTAGGGAGGAGCTTTTTCGTGGATT |
| 7^a^ | 223.3 | Allele-1 | GAAGGTGACCAAGTTCATGCTTTTTCGTGGATTCGCCGTCG |
|  |  | Allele-2 | GAAGGTCGGAGTCAACGGATTGCTTTTTCGTGGATTCGCCGTCA |
|  |  | Reverse | CGGAGCTCTAGATTGATTCTGCTCAA |
| 8 | 247.8 | Allele-1 | GAAGGTGACCAAGTTCATGCTCTGTGATGGCAGACGGCAAAG |
|  |  | Allele-2 | GAAGGTCGGAGTCAACGGATTGTCTGTGATGGCAGACGGCAAAA |
|  |  | Reverse | CCTCCGGTGTCAATCACTATAGGAA |
| 9 | 266.4 | Allele-1 | GAAGGTGACCAAGTTCATGCTAACAAAGATATATTTTTCTTCAATCTTGTTCC |
|  |  | Allele-2 | GAAGGTCGGAGTCAACGGATTGAACAAAGATATATTTTTCTTCAATCTTGTTCT |
|  |  | Reverse | CACCAAGCTGAAATGTCAATCTTCACAAT |
| 10 | 285.8 | Allele-1 | GAAGGTGACCAAGTTCATGCTACCACCGCTTGAAAAAGTATTCGC |
|  |  | Allele-2 | GAAGGTCGGAGTCAACGGATTCTACCACCGCTTGAAAAAGTATTCGT |
|  |  | Reverse | CTGAGCGGTTGTACTAAGTTGGACTT |
| 10^a^ | 285.8 | Allele-1 | GAAGGTGACCAAGTTCATGCTGCGGTTGTACTAAGTTGGACTTTTTG |
|  |  | Allele-2 | GAAGGTCGGAGTCAACGGATTAGCGGTTGTACTAAGTTGGACTTTTTA |
|  |  | Reverse | GGCCTACCACCGCTTGAAAAAGTAT |
| 11 | 325.9 | Allele-1 | GAAGGTGACCAAGTTCATGCTGCGCCGCCTTGAACTCCAG |
|  |  | Allele-2 | GAAGGTCGGAGTCAACGGATTCAGCGCCGCCTTGAACTCCAA |
|  |  | Reverse | CCTGGCCTCTGCGGCGGAT |
| 12 | 375.2 | Allele-1 | GAAGGTGACCAAGTTCATGCTGAAGGTCTATTTTCACAGAGTCAGC |
|  |  | Allele-2 | GAAGGTCGGAGTCAACGGATTGGAAGGTCTATTTTCACAGAGTCAGT |
|  |  | Reverse | CCGGCAACGGCGCGAGAAAAAT |
| 13 | 395.2 | Allele-1 | GAAGGTGACCAAGTTCATGCTGTGTTACAAACCTTACCCCCTTAG |
|  |  | Allele-2 | GAAGGTCGGAGTCAACGGATTGGTGTTACAAACCTTACCCCCTTAA |
|  |  | Reverse | TGGCAATCTCGAGGACGAGATTCTT |
| 13^a^ | 395.2 | Allele-1 | GAAGGTGACCAAGTTCATGCTCAATCTCGAGGACGAGATTCTTC |
|  |  | Allele-2 | GAAGGTCGGAGTCAACGGATTGCAATCTCGAGGACGAGATTCTTT |
|  |  | Reverse | GGGTGTTACAAACCTTACCCCCTTA |
| 14 | 441.6 | Allele-1 | GAAGGTGACCAAGTTCATGCTGCCCTCTGGCCTCGAAAGC |
|  |  | Allele-2 | GAAGGTCGGAGTCAACGGATTGGCCCTCTGGCCTCGAAAGT |
|  |  | Reverse | CAGGCGGCAGAACTCCGGAAA |
| 15 | 463.7 | Allele-1 | GAAGGTGACCAAGTTCATGCTCGGTCAATGTTGGCTTGATGAAG |
|  |  | Allele-2 | GAAGGTCGGAGTCAACGGATTGCGGTCAATGTTGGCTTGATGAAA |
|  |  | Reverse | GGTTGCACGGGTCGGGCGTA |

^a^Redesigned due to failures.
